# Supplementary material for: SARS-CoV-2 B.1.1.7 (alpha) and B.1.351 (beta) variants induce pathogenic patterns in K18-hACE2 transgenic mice distinct from early strains
Source: Nat Commun. 2021 Nov 12;12:6559. doi: 10.1038/s41467-021-26803-w (PMC8589842; doi:10.1038/s41467-021-26803-w)
Supplement: Supplementary file 1 — Supplementary Information [file 41467_2021_26803_MOESM1_ESM.pdf]

Supplementary Information

SARS-CoV-2 B.1.1.7 (alpha) and B.1.351 (beta) variants induce pathogenic patterns in K18-hACE2 transgenic mice distinct from early strains

Peter Radvak[1,5], Hyung Joon Kwon[1,5], Martina Kosikova[1,5], Uriel Ortega-Rodriguez[1,5], Ruoxuan Xiang[2], Je-Nie Phue[3], Rong-Fong Shen[3], James Rozzelle[4], Neeraj Kapoor[4], Taylor Rabara[4], Jeff Fairman[4], Hang Xie[1*]

[1]Laboratory of Pediatric and Respiratory Viral Diseases, Division of Viral Products, Office of Vaccines Research and Review, [2]Division of Biostatistics, Office of Biostatistics and Epidemiology, [3]Facility for Biotechnology Resources, Center for Biologics Evaluation and Research, United States Food and Drug Administration, Silver Spring, Maryland, USA; [4]Vaxcyte, Inc., Foster City, California, USA.

[5]These authors contributed equally: Peter Radvak, Hyung Joon Kwon, Martina Kosikova, Uriel Ortega-Rodriguez.

[*]Corresponding author: Hang Xie (Email: Hang.Xie@fda.hhs.gov)

Supplementary information includes:

- Six supplementary figures.
- One supplementary table.

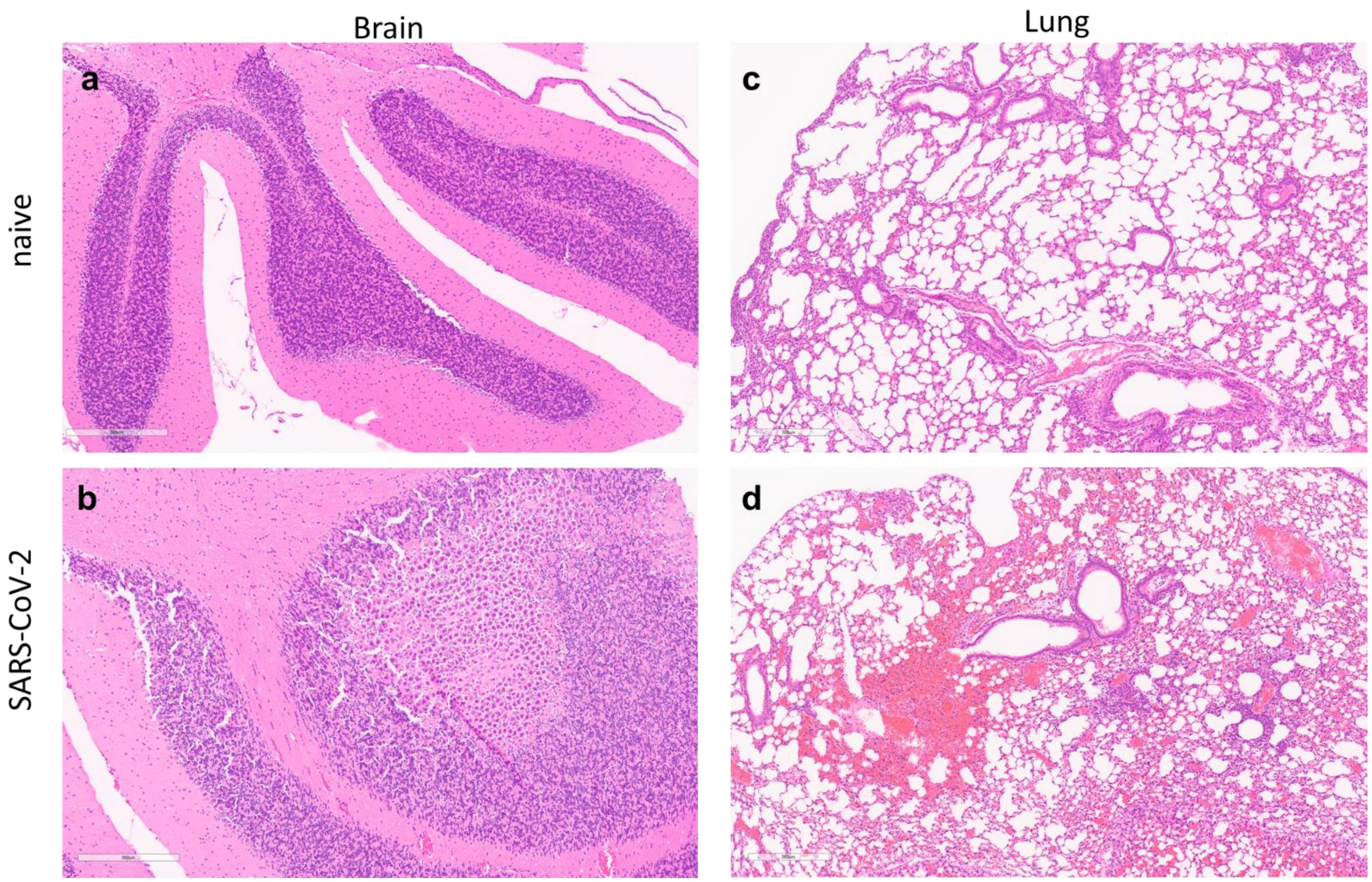

Supplementary Figure 1. Histopathology of lung and brain of K18-hACE2 mice after SARS-CoV-2 infection. Lungs and brains were harvested from K18-hACE2 mice at day 6 post infection (dpi 6) of hCoV-19/South Africa/KRISP-EC-K005321/2020 (SA) of lineage B.1.351 (10^2 $TCID_{50}$/mouse). Representative images of hematoxylin and eosin staining of lung (a & b) and brain (c & d) tissues from naïve or infected K18-hACE2 mice (n=6 mice/group) are shown. Scale bars, 300 µm.

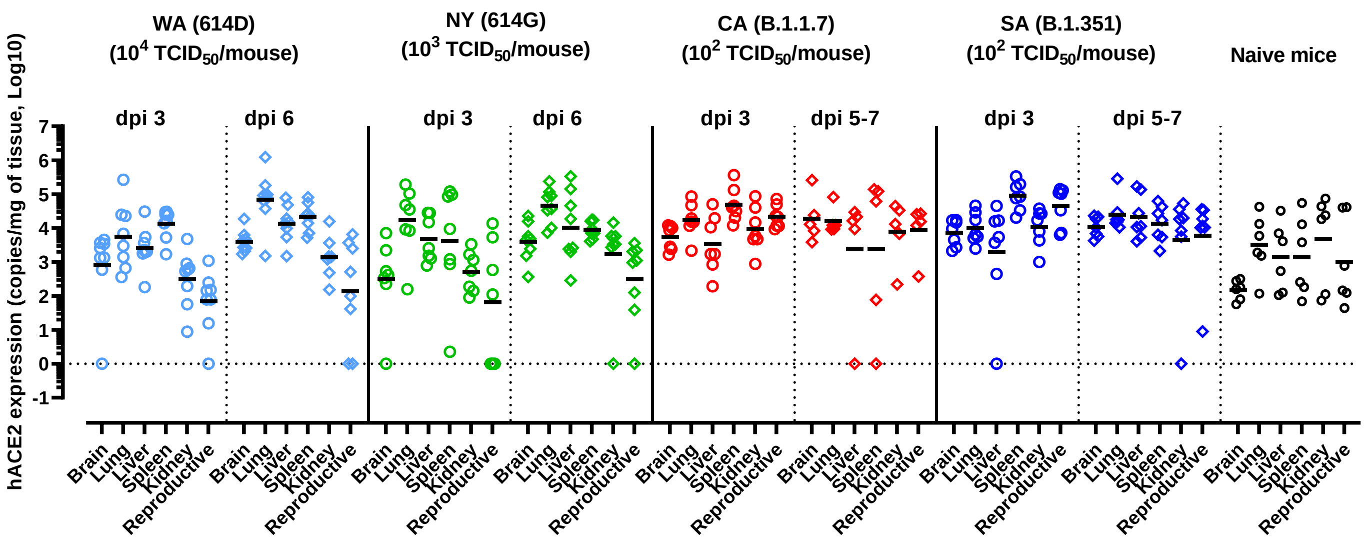

Supplementary Figure 2. hACE2 expression in various organs of K18-hACE2 mice after infections of SARS-CoV-2 and variants. K18-hACE2 mice of both sexes (approximately 1:1 ratio) were infected intranasally with USA-WA1/2020 (WA) of lineage A bearing 614D (10^4 TCID$_{50}$/mouse), New York-PV09158/2020 (NY) of lineage B.1.3 bearing 614G (10^3 TCID$_{50}$/mouse), USA/CA_CDC_5574/2020 (CA) of lineage B.1.1.7 (10^2 TCID$_{50}$/mouse) or hCoV-19/South Africa/KRISP-EC-K005321/2020 (SA) of lineage B.1.351 (10^2 TCID$_{50}$/mouse). hACE2 expression in brain, lung, liver, spleen, kidney, and reproductive organs (ovary or seminal vesicles) at indicated days post infection (dpi) were measured by RT-qPCR. Data are expressed as mean ± s.e.m of 5-8 mice/time point/group. Short lines represent geometric means and dotted horizontal line indicates the limit of detection.

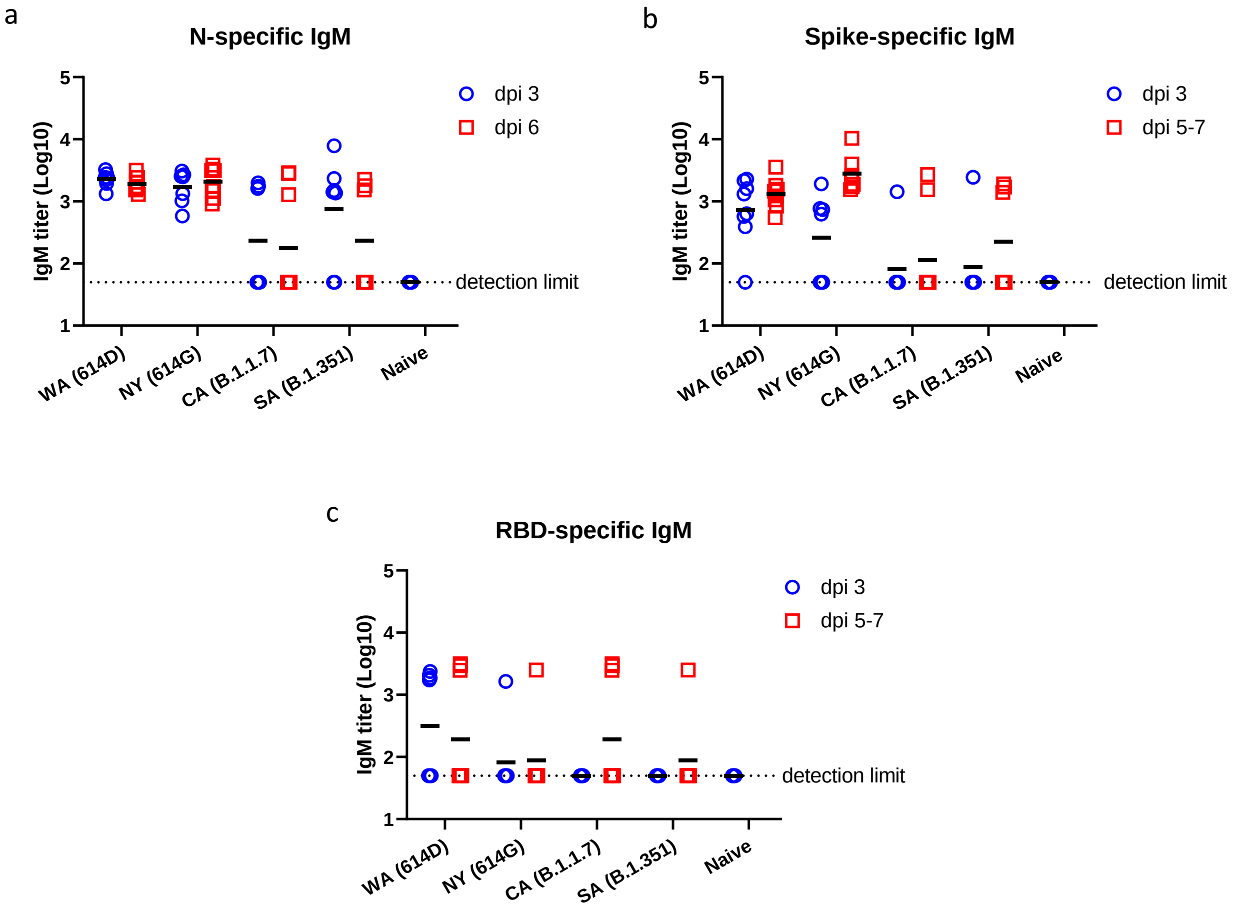

Supplementary Figure 3. Detection of virus-specific IgM in K18-hACE2 after infections of SARS-CoV-2 and variants. K18-hACE2 mice of both sexes (approximately 1:1 ratio) were infected intranasally with USA-WA1/2020 (WA) of lineage A bearing 614D (10^4 $TCID_{50}$/mouse), New York-PV09158/2020 (NY) of lineage B.1.3 bearing 614G (10^3 $TCID_{50}$/mouse), USA/CA_CDC_5574/2020 (CA) of lineage B.1.1.7 (10^2 $TCID_{50}$/mouse) or hCoV-19/South Africa/KRISP-EC-K005321/2020 (SA) of lineage B.1.351 (10^2 $TCID_{50}$/mouse). Sera were collected at indicated days post infection (dpi) for IgM ELISA titers specific for SARS-CoV-2 (a) nucleocapsid (N), (b) spike, or (c) receptor binding domain (RBD). Short lines represent geometric means of 6-9 mice/time point/group and dotted horizontal lines indicate the limit of detection.

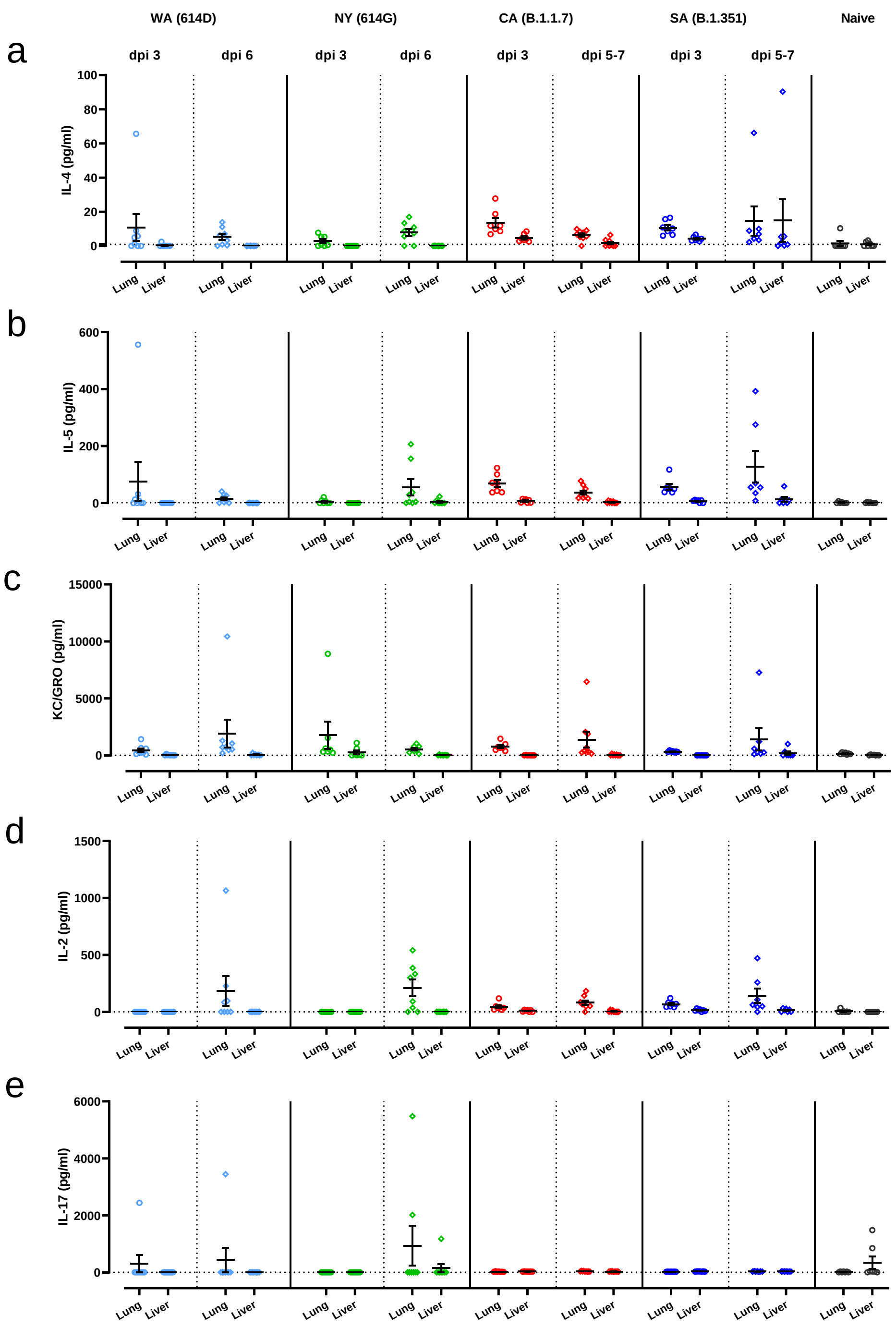

Supplementary Figure 4. Additional proinflammatory cytokines induced by infections of SARS-CoV-2 or variants. K18-hACE2 mice of both sexes (approximately 1:1 ratio) were infected intranasally with USA-WA1/2020 (WA) of lineage A bearing 614D, New York-PV09158/2020 (NY) of lineage B.1.3 bearing 614G, USA/CA_CDC_5574/2020 (CA) of lineage B.1.1.7 or hCoV-19/South Africa/KRISP-EC-K005321/2020 (SA) of lineage B.1.351 at indicated doses in an ABSL-3 biocontainment. Additional mouse cytokines in lung and liver homogenates harvested at indicated days post infection (dpi) were measured (n=7-9 mice/time point/group), including (a) IL-4; (b) IL-5; (c) KC/GRO; (d) IL-2; and (e) IL-17. Mean ± s.e.m are shown and dotted horizontal lines indicate the limit of detection.

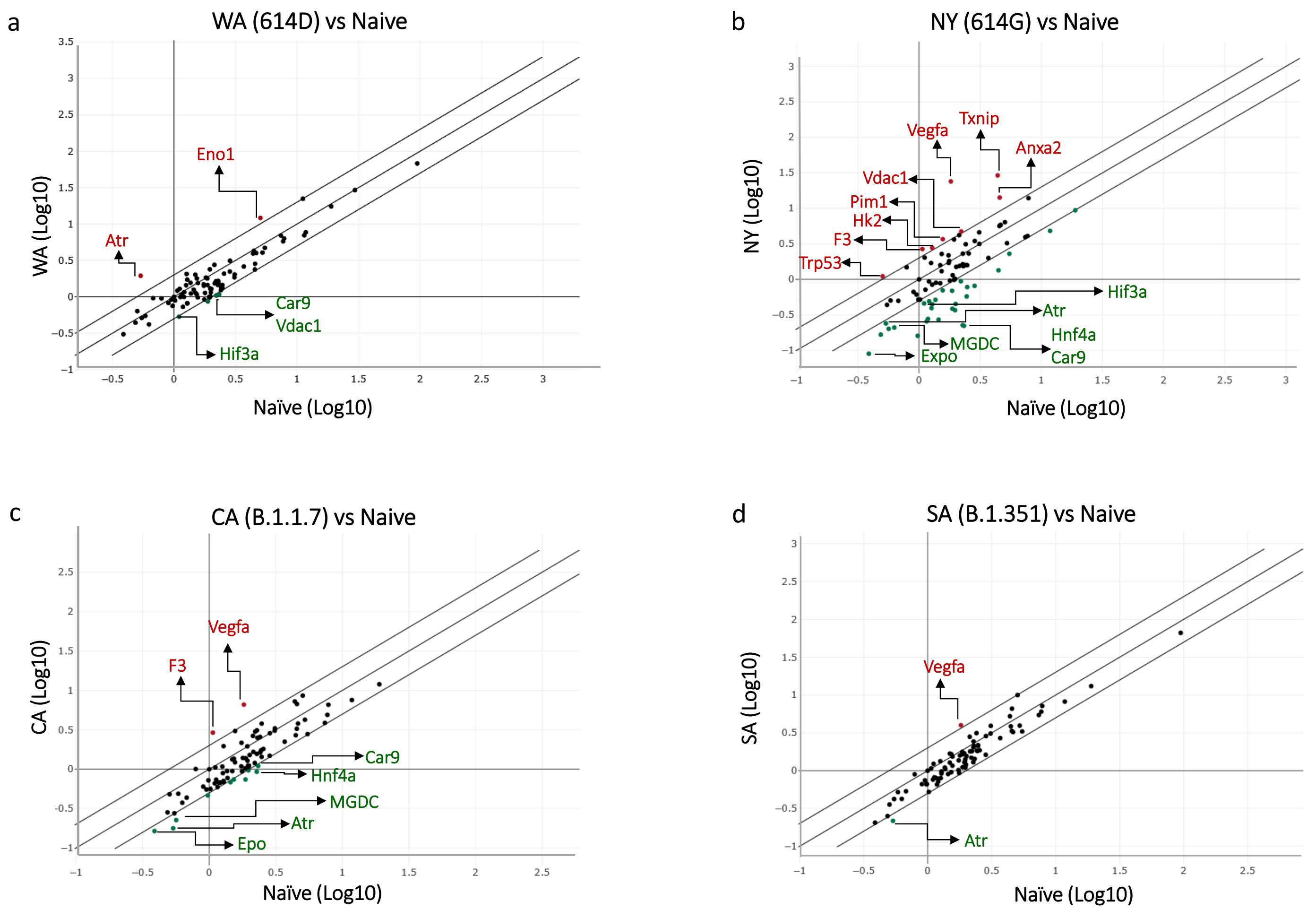

Supplementary Figure 5. The correlation scatter plot of pulmonary gene expression of hypoxia signaling pathway after SARS-CoV-2 infections. K18-hACE2 mice of both sexes (approximately 1:1 ratio) were infected intranasally with USA-WA1/2020 (WA) of lineage A bearing 614D (10^4 $TCID_{50}$/mouse), New York-PV09158/2020 (NY) of lineage B.1.3 bearing 614G (10^3 $TCID_{50}$/mouse), USA/CA_CDC_5574/2020 (CA) of lineage B.1.1.7 (10^2 $TCID_{50}$/mouse) or hCoV-19/South Africa/KRISP-EC-K005321/2020 (SA) of lineage B.1.351 (10^2 $TCID_{50}$/mouse). Hypoxia signaling pathway PCR array was performed using RNA extracted from lung homogenates of naïve or infected mice at day 3 post infection (n= 4-5 mice/group). The average fold changes of individual genes induced by SARS-CoV-2 infection, including (a) WA (614D), (b) NY (614G), (c) CA (B.1.1.7) and (d) SA (B.1.351) vs naïve mice are presented in the correlation scatter plots. Genes beyond the 2-fold change lines are marked by red (upregulation) or green (downregulation) dots. Typical genes that involve in the hypoxia signaling pathway are labeled.

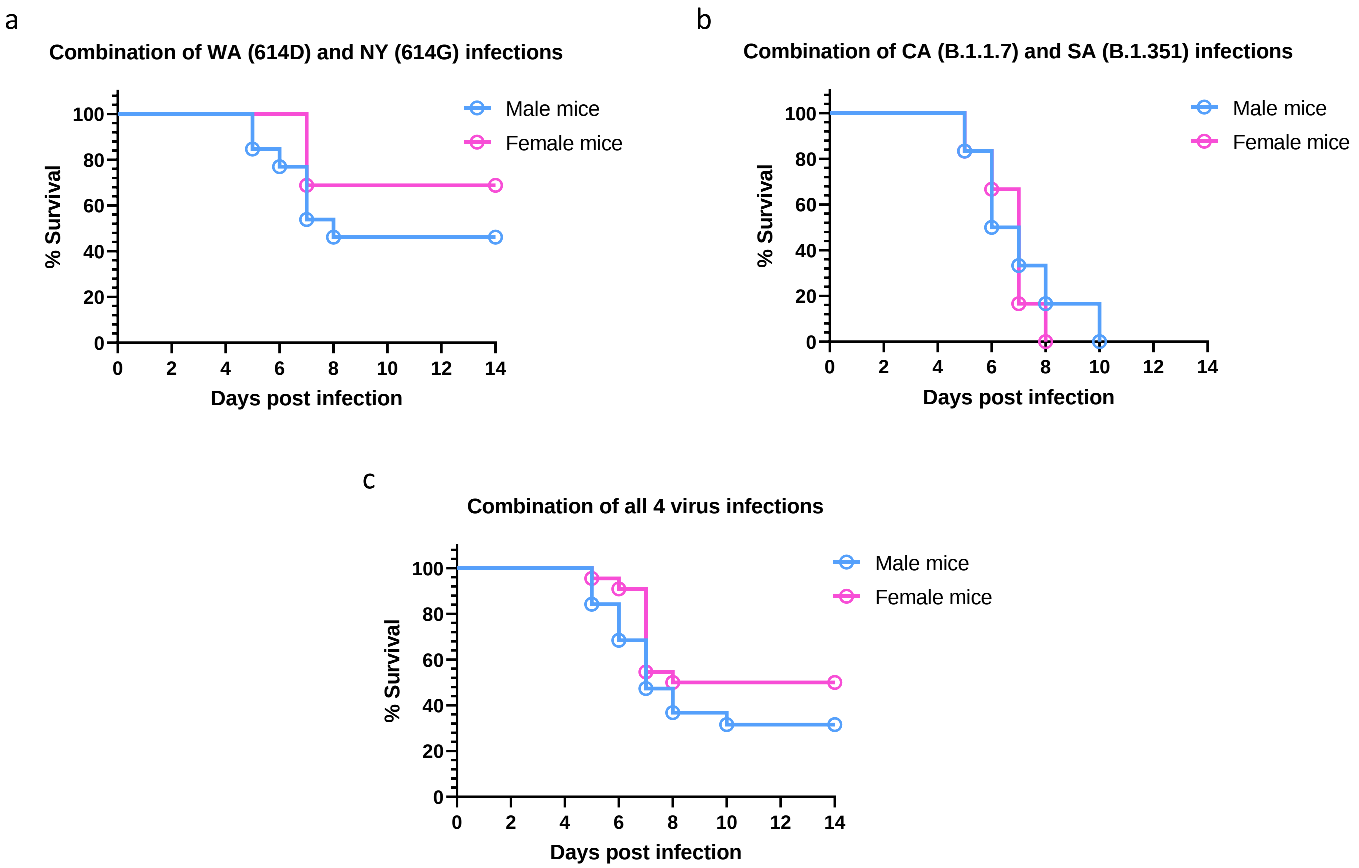

Supplementary Figure 6. Sex-biased difference in the survivals of K18-hACE2 mice after SARS-CoV-2 infections. K18-hACE2 mice of both sexes (approximately 1:1 ratio) were infected intranasally with USA-WA1/2020 (WA) of lineage A bearing 614D, New York-PV09158/2020 (NY) of lineage B.1.3 bearing 614G, USA/CA_CDC_5574/2020 (CA) of lineage B.1.1.7 or hCoV-19/South Africa/KRISP-EC-K005321/2020 (SA) of lineage B.1.351 at indicated doses shown in Figure 1c. The % survivals of infected mice shown in Figure 1c were reanalyzed according to the sexes. (a) the combined survival of female and male mice after infection with WA (614D) or NY (614G) (n= 15-16 mice/sex/group); (b) the combined survivals of female and male mice after infection with CA (B.1.1.7) or SA (B.1.351) (n=6 mice/sex/group); (c) the combined survivals of all female and male mice after 4 virus infections (n= 21-22 mice/sex/group).

Supplementary Table 1. Regulation of the hypoxia pathway transcriptional genes in the lungs of K18-hACE2 transgenic mice at day 3 post SARS-CoV-2 infection*.

Gene Symbol	WA (614D)		NY (614G)		CA (B.1.1.7)		SA (B.1.351)	
	Fold change	p value	Fold change	p value	Fold change	p value	Fold change	p value
Adm	0.77	0.269308	0.16	0.016381	0.48	0.122498	0.68	0.378990
Adora2b	0.93	0.621161	0.22	0.023513	0.51	0.070354	0.70	0.174827
Aldoa	0.81	0.401227	1.77	0.579180	0.84	0.474676	0.91	0.704539
Angptl4	0.71	0.172278	0.72	0.096828	0.77	0.139424	0.84	0.278878
Ankrd37	1.63	0.005757	0.31	0.010772	0.70	0.205102	0.63	0.138468
Anxa2	0.62	0.337070	3.12	0.356016	1.48	0.915868	1.45	0.904911
Apex1	0.78	0.295050	0.28	0.035834	0.52	0.141486	0.57	0.141873
Arnt	0.82	0.321351	0.91	0.570315	0.71	0.240713	0.73	0.257010
Atr	3.64	0.021031	0.45	0.377243	0.33	0.187373	0.41	0.198955
Bhlhe40	0.94	0.869219	0.86	0.893131	0.84	0.270078	0.89	0.461663
Blm	0.87	0.441187	0.19	0.020108	0.47	0.164501	0.63	0.161608
Bnip3	0.86	0.455653	0.42	0.102522	0.51	0.141487	0.60	0.175286
Bnip3l	0.94	0.562240	0.53	0.226751	0.52	0.173283	0.74	0.426029
Btg1	1.10	0.912006	1.22	0.921141	1.00	0.714312	1.01	0.786513
Car9	0.46	0.003799	0.10	0.001146	0.47	0.038216	0.62	0.218879
Ccng2	0.75	0.183647	0.43	0.055550	0.55	0.096137	0.67	0.174187
Cops5	0.87	0.489682	0.51	0.110915	0.55	0.114167	0.51	0.098959
Ctsa	0.65	0.183054	1.01	0.760932	1.06	0.841752	1.26	0.634928
Ddit4	0.52	0.113173	1.03	0.944726	0.53	0.159299	0.53	0.155666
Dnajc5	0.66	0.215296	0.52	0.162531	0.54	0.159433	0.72	0.265252
Edn1	0.91	0.488113	0.74	0.295416	0.67	0.195337	0.74	0.271400
Egln1	1.16	0.779340	1.82	0.506844	0.95	0.573438	1.11	0.794826
Egln2	0.64	0.111181	0.72	0.274135	0.69	0.225407	0.90	0.610598
Egr1	0.73	0.315597	1.01	0.850701	1.37	0.534166	1.07	0.867169
Eif4ebp1	0.96	0.714405	0.41	0.078320	0.56	0.065642	0.60	0.074379
Eno1	2.41	0.791075	1.27	0.297333	1.71	0.442002	1.98	0.524637
Epo	0.79	0.277921	0.23	0.043037	0.42	0.105921	0.53	0.138256
Ero1l	1.15	0.816237	1.35	0.423492	0.86	0.434190	1.06	0.959584
F10	0.96	0.517411	0.53	0.142132	0.73	0.258602	0.67	0.219893
F3	1.14	0.933498	2.51	0.143504	2.74	0.138802	1.01	0.712684
Fos	0.53	0.153161	0.64	0.485989	1.53	0.489565	0.86	0.486594
Gbe1	0.83	0.435986	0.78	0.427685	0.56	0.252594	0.65	0.310389
Gpi1	0.52	0.133429	1.24	0.977516	0.72	0.336094	0.74	0.607497
Gys1	0.54	0.088784	0.23	0.014333	0.71	0.330067	0.74	0.487989
Hif1a	1.35	0.807847	0.59	0.359503	0.64	0.342271	0.86	0.572808
Hif1an	0.96	0.642490	0.64	0.150803	0.58	0.117256	0.78	0.336065
Hif3a	0.49	0.001406	0.42	0.003423	0.60	0.212977	0.69	0.634791
Hk2	0.63	0.114526	2.17	0.251354	1.53	0.278265	0.74	0.296431
Hmox1	0.88	0.391368	0.63	0.208015	0.65	0.258955	0.72	0.242833
Hnf4a	0.65	0.040780	0.10	0.000853	0.41	0.037066	0.57	0.017748
Ier3	0.68	0.132246	0.67	0.196062	1.09	0.929228	0.92	0.732974
Igfbp3	1.22	0.427641	0.51	0.053710	0.57	0.093931	0.67	0.129406
Jmjd6	0.63	0.029778	0.54	0.021902	0.78	0.466171	0.89	0.662104
Ldha	0.92	0.542851	0.50	0.057768	0.63	0.154154	0.69	0.155281
Lgals3	0.81	0.321213	0.97	0.780335	1.24	0.780455	0.78	0.367502
Lox	0.85	0.417355	1.25	0.678904	0.62	0.235886	0.59	0.211033
Map3k1	1.41	0.723241	0.74	0.506460	0.64	0.281945	0.79	0.409669
Met	0.78	0.305263	0.21	0.042116	0.40	0.108406	0.59	0.189817
Mif	0.65	0.063105	0.41	0.022440	0.64	0.133491	0.70	0.135786
Mmp9	0.59	0.097565	0.31	0.104277	0.58	0.194994	0.88	0.513771
Mxi1	0.81	0.357518	0.65	0.225659	0.56	0.162466	0.77	0.370764
Nampt	1.29	0.562521	1.97	0.091137	1.24	0.653509	1.32	0.519812
Ncoa1	1.34	0.141190	0.66	0.044033	0.53	0.018609	0.76	0.102682
Ndrg1	0.71	0.173677	0.85	0.617098	0.48	0.079650	0.63	0.151920
Nfkb1	0.77	0.358910	1.06	0.805571	0.90	0.533435	1.13	0.906420
Nos3	0.64	0.174918	0.35	0.067778	0.55	0.186433	0.72	0.364739
Odc1	0.77	0.311241	0.62	0.373522	0.81	0.415789	0.75	0.369219
P4ha1	1.17	0.065483	1.49	0.131666	0.89	0.233690	1.08	0.525909
P4hb	0.51	0.198874	1.30	0.900770	1.07	0.738565	1.30	0.804818

Pdk1	1.15	0.901620	0.38	0.104645	0.70	0.345338	0.81	0.541256
Per1	0.52	0.045221	1.89	0.130082	1.01	0.851469	0.88	0.468096
Pfkfb3	0.62	0.182294	0.23	0.067607	0.57	0.230130	0.55	0.175394
Pfkfb4	0.50	0.031108	0.51	0.047339	0.66	0.190313	0.67	0.135363
Pfkl	0.64	0.110442	0.74	0.220654	0.75	0.258039	0.77	0.257403
Pfkp	0.55	0.334647	0.66	0.310140	0.68	0.320868	0.77	0.407603
Pgam1	0.75	0.231790	0.53	0.125501	0.64	0.214433	0.78	0.345939
Pgf	0.51	0.120932	0.19	0.070372	0.49	0.168395	0.56	0.236237
Pgk1	0.86	0.428425	1.26	0.661193	0.82	0.417633	0.70	0.275430
Pim1	0.99	0.856567	2.35	0.034723	1.96	0.097982	0.98	0.820961
Pkm	0.60	0.220874	1.48	0.662689	0.99	0.737554	1.00	0.818175
Plau	0.91	0.494848	0.34	0.034250	0.58	0.093728	0.52	0.071724
Rbpj	0.96	0.629616	0.30	0.065947	0.60	0.172620	0.86	0.474724
Ruvbl2	0.46	0.012554	0.61	0.048695	0.73	0.213056	0.88	0.401681
Serpine1	1.20	0.681532	1.87	0.137648	1.27	0.572249	1.13	0.906491
Slc16a3	0.58	0.046063	0.23	0.011951	0.56	0.077815	0.60	0.126919
Slc2a1	0.63	0.069858	0.45	0.041675	0.78	0.352314	0.75	0.213201
Slc2a3	0.67	0.214005	0.33	0.124332	0.60	0.216171	0.68	0.306115
Tfrc	0.96	0.602276	0.37	0.039797	0.55	0.102970	0.95	0.665389
Tpi1	0.71	0.122356	0.54	0.056810	0.61	0.087930	0.73	0.188019
Trp53	1.27	0.765312	2.20	0.115682	0.96	0.664814	0.71	0.305228
Txnip	0.90	0.510364	6.63	0.041210	1.65	0.653311	1.20	0.953711
Usf2	0.94	0.493867	0.80	0.767696	0.50	0.141499	0.77	0.475304
Vdac1	0.47	0.154186	2.14	0.219265	1.36	0.717820	0.82	0.449406
Vegfa	1.01	0.617330	13.18	0.018136	3.63	0.169917	2.20	0.039536
Actb	0.99	0.512510	4.08	0.269820	1.78	0.999793	1.54	0.764659
B2m	2.00	0.764139	9.03	0.049525	6.29	0.118950	5.12	0.220735
Gapdh	0.72	0.174689	0.18	0.025330	0.57	0.184862	0.70	0.236111
Gusb	1.00	0.393781	1.00	0.450779	1.00	0.934536	1.00	0.307071
Hsp90ab1	0.61	0.292158	3.29	0.197661	1.14	0.694994	1.11	0.886919

* Lung homogenates of K18-hACE2 mice (n=4-5 mice/group) at day 3 post infection of USA-WA1/2020 (WA) of lineage A bearing 614D (10^4 $TCID_{50}$/mouse), New York-PV09158/2020 (NY) of lineage B.1.3 bearing 614G (10^3 $TCID_{50}$/mouse), USA/CA_CDC_5574/2020 (CA) of lineage B.1.1.7 (10^2 $TCID_{50}$/mouse) or hCoV-19/South Africa/KRISP-EC-K005321/2020 (SA) of lineage B.1.351 (10^2 $TCID_{50}$/mouse) were subjected to the Hypoxia signaling pathway PCR array. Fold changes of individual genes over naïve mice are reported.
