# Supplementary material for: SARS-CoV-2 B.1.1.7 (alpha) and B.1.351 (beta) variants induce pathogenic patterns in K18-hACE2 transgenic mice distinct from early strains
Source: Nat Commun. 2021 Nov 12;12:6559. doi: 10.1038/s41467-021-26803-w (PMC8589842; doi:10.1038/s41467-021-26803-w)
Supplement: Supplementary file 2 — Reporting Summary [file 41467_2021_26803_MOESM2_ESM.pdf]

nature portfolio

Corresponding author(s): Hang Xie

Last updated by author(s): Oct 12, 2021

Reporting Summary

Nature Portfolio wishes to improve the reproducibility of the work that we publish. This form provides structure for consistency and transparency in reporting. For further information on Nature Portfolio policies, see our Editorial Policies and the Editorial Policy Checklist.

Statistics

For all statistical analyses, confirm that the following items are present in the figure legend, table legend, main text, or Methods section.

n/a	Confirmed	
[ ]	[x]	The exact sample size (*n*) for each experimental group/condition, given as a discrete number and unit of measurement
[ ]	[x]	A statement on whether measurements were taken from distinct samples or whether the same sample was measured repeatedly
[ ]	[x]	The statistical test(s) used AND whether they are one- or two-sided<br>*Only common tests should be described solely by name; describe more complex techniques in the Methods section.*
[ ]	[x]	A description of all covariates tested
[x]	[ ]	A description of any assumptions or corrections, such as tests of normality and adjustment for multiple comparisons
[ ]	[x]	A full description of the statistical parameters including central tendency (e.g. means) or other basic estimates (e.g. regression coefficient) AND variation (e.g. standard deviation) or associated estimates of uncertainty (e.g. confidence intervals)
[ ]	[x]	For null hypothesis testing, the test statistic (e.g. *F*, *t*, *r*) with confidence intervals, effect sizes, degrees of freedom and *P* value noted<br>*Give P values as exact values whenever suitable.*
[x]	[ ]	For Bayesian analysis, information on the choice of priors and Markov chain Monte Carlo settings
[x]	[ ]	For hierarchical and complex designs, identification of the appropriate level for tests and full reporting of outcomes
[ ]	[x]	Estimates of effect sizes (e.g. Cohen's *d*, Pearson's *r*), indicating how they were calculated

Our web collection on statistics for biologists contains articles on many of the points above.

Software and code

Policy information about availability of computer code

Data collection	Data were collected using Microsoft Excel (Version 2102, Microsoft Office 365) or built-in software Wallac 1420 Workstation (Version 3 Revision 4) in PerkinElmer Victor V multilabel reader, MSD Discovery Workbench 4.0.12 (LSR_4_0_12) in MESO QuickPlex SQ 120 imager & MxPro (Version 3.0) in Stratagene MX3000p qPCR system. Aperio ImageScope DX clinical viewing software version 12.4.3.5008.
Data analysis	R version 4.0.3 (https://www.r-project.org/) and Prism 8.4.3 (GraphPad, San Diego, CA) were used for statistical analyses. Hypoxia pathway gene expression was calculated using Qiagen web-based GeneGlobe data analysis tool (https://geneglobe.qiagen.com/ua/analyze).

For manuscripts utilizing custom algorithms or software that are central to the research but not yet described in published literature, software must be made available to editors and reviewers. We strongly encourage code deposition in a community repository (e.g. GitHub). See the Nature Portfolio guidelines for submitting code & software for further information.

Data

Policy information about availability of data

All manuscripts must include a data availability statement. This statement should provide the following information, where applicable:

- Accession codes, unique identifiers, or web links for publicly available datasets
- A description of any restrictions on data availability
- For clinical datasets or third party data, please ensure that the statement adheres to our policy

Source data are provided with this paper. PCR array data that support the findings of this study have also been deposited in NCBI GEO with the accession number GSE183621 (https://www.ncbi.nlm.nih.gov/geo/query/acc.cgi?acc=GSE183621).The DNA sequences encoding the full-length S (residues 1-1213) and RBD (residues 319-541) of WA are obtained from GenBank: MN985325.1.

Field-specific reporting

Please select the one below that is the best fit for your research. If you are not sure, read the appropriate sections before making your selection.

- [x] Life sciences
- [ ] Behavioural & social sciences
- [ ] Ecological, evolutionary & environmental sciences

For a reference copy of the document with all sections, see nature.com/documents/nr-reporting-summary-flat.pdf

Life sciences study design

All studies must disclose on these points even when the disclosure is negative.

Sample size	Sample size was determined based on previous experience in investigating viral pathogenesis in mice. In most experiments 7-9 K18-hACE2 transgenic mice per group were used, which was consistent with similar studies as published by other labs. The exact sample size for each experiment is indicated in individual figure legends.
Data exclusions	No data were excluded.
Replication	All animal experiments were repeated at least twice. Serum microneutralization and antibody titers of individual mice were assayed 2-3 times. Tissue pathology (n=6 mice/group). All attempts at replication were successful.
Randomization	The animals of both sexes were randomly grouped and allocated in each experiment.
Blinding	Blinding was not applicable, because SARS-CoV-2 is high risk pathogen and all related works are restricted in ABSL3. Only approved laboratory staff with ABSL3 clearance have access to SARS-CoV-2 and they must know what pathogen they work on in compliance with the approved ABSL3 protocols.

Reporting for specific materials, systems and methods

We require information from authors about some types of materials, experimental systems and methods used in many studies. Here, indicate whether each material, system or method listed is relevant to your study. If you are not sure if a list item applies to your research, read the appropriate section before selecting a response.

Materials & experimental systems

n/a	Involved in the study	
[ ]	[x]	Antibodies
[ ]	[x]	Eukaryotic cell lines
[x]	[ ]	Palaeontology and archaeology
[ ]	[x]	Animals and other organisms
[x]	[ ]	Human research participants
[x]	[ ]	Clinical data
[x]	[ ]	Dual use research of concern

Methods

n/a	Involved in the study	
[x]	[ ]	ChIP-seq
[x]	[ ]	Flow cytometry
[x]	[ ]	MRI-based neuroimaging

Antibodies

Antibodies used	Peroxidase-conjugated goat anti-mouse IgM heavy chain (Southern Biotech Cat No. 1021-05): 1:4000.<br>Peroxidase-conjugated goat anti-mouse IgG (H+L) (Invitrogen Cat No. A24518): 1:2000.<br>Peroxidase-conjugated goat anti-rabbit IgG (H+L) (Invitrogen Cat No. 31460): 1:2000.<br>In-house developed rabbit polyclonal antibody specific for SARS-CoV-2 E/M/N: 1:1000.
Validation	The validation of commercial antibodies can be found from the manufacturers' websites.<br>The rabbit polyclonal antibodies specific for SARS-CoV-2 E/M/N developed in house have been validated using human COVID-19 convalescent sera and have been used to establish the 1st World Health Organization International Standard and Reference Panel for anti-SARS-CoV-2 antibody (https://www.who.int/publications/m/item/WHO-BS-2020.2403).

Eukaryotic cell lines

Policy information about cell lines

Cell line source(s)	Vero E6 (CRL-1586) was obtained from ATCC. Expi293F was obtained from ThermoFisher (#A14635).
Authentication	Vero E6 and Expi293F are commercially available. Only limited passages were performed after Vero E6 and Expi293F were received and recovered.

Mycoplasma contamination	Vero E6 and Expi293F were negative for mycoplasma contamination.
Commonly misidentified lines (See ICLAC register)	No commonly misidentified cell lines were used.

Animals and other organisms

Policy information about studies involving animals; ARRIVE guidelines recommended for reporting animal research

Laboratory animals	SPF Hemizygous B6.Cg-Tg(K18-ACE2)2Prlmn/J (K18-hACE) female mice (JAX Stock No. 034860) and noncarrier c57BL/6J male mice (JAX Stock No. 000664) were obtained from Jackson Laboratory and were mated to maintain live colonies at the ABSL2 facility of FDA White Oak Vivarium. Only hACE2 (+) offspring as confirmed by genotyping were retained and were used at 8-10 weeks old for the experiments.
Wild animals	No wild animals were used.
Field-collected samples	No field-collected samples.
Ethics oversight	The animal study protocol was approved by the FDA White Oak Animal Program Animal Care and Use Committee

Note that full information on the approval of the study protocol must also be provided in the manuscript.
